# Alcohol exposure significantly influences gene expression in the hypothalamus, highlighting complex links with gonadotropin-releasing hormone signaling and thyroid hormone production in adolescent mice

**DOI:** 10.1186/s12920-025-02235-z

**Published:** 2025-10-07

**Authors:** Guangtao Sun, Yujing Peng, Chenxu Hu, Yifan Zheng, Yu Cheng, Xunzhong Qi, Yuling Jin

**Affiliations:** Department of Neurology, The First Affiliated Hospital of Jiamusi University, Jiamusi, People’s Republic of China

**Keywords:** Alcohol use disorder, Hypothalamus, Transcriptome sequencing, GnRH signaling pathway, Thyroid hormone synthesis

## Abstract

**Objective:**

Hypothalamic dysfunction occurs in alcohol use disorder (AUD). Here, we investigated the effects of alcohol exposure on hypothalamic gene expression in mice, and examined the role of the hypothalamus in AUD pathogenesis.

**Methods:**

An alcohol exposure model was constructed in male C57BL/6 mice using the two-bottle drinking method. Transcriptome sequencing was used to analyze differential gene expression in the hypothalamus of alcohol exposure model and control mice. Gene Ontology (GO) and Kyoto Encyclopedia of Genes and Genomes (KEGG) analyses of the differentially expressed genes were performed. In addition, real-time quantitative PCR was used to verify the differential expression of genes.

**Results:**

We identified 225 differentially expressed genes by transcriptome sequencing, of which 64 showed increased expression and 161 decreased expression. GO enrichment analysis showed highest enrichment for developmental process terms. KEGG enrichment analysis showed highest enrichment for the gonadotropin-releasing hormone (GnRH) signaling pathway. PCR validation showed reduced expression of *Prkcd*, *Ptk2b*, and *Adcy1* in the alcohol group compared with the control group, consistent with the sequencing results. The thyroid hormone synthesis pathway was significantly enriched, and PCR results showed that expression of *Adcy1*, *Gpx2*, and *Ttr* were decreased in the alcohol group compared with the control group, which was consistent with the sequencing results.

**Conclusion:**

Alcohol exposure in mice modulates the expression of genes associated with hypothalamic GnRH signaling pathway and thyroid hormone synthesis pathway. Expression of GnRH signaling pathway genes, *Prkcd* and *Ptk2b*, and of thyroid hormone synthesis pathway genes, *Gpx2*, *Ttr*, and *Adcy1*, was decreased. Our findings indicate that alcohol exposure is associated with altered expression of these genes, which may be relevant to the pathophysiology of AUD.

**Supplementary Information:**

The online version contains supplementary material available at 10.1186/s12920-025-02235-z.

## Introduction

Alcohol use disorder (AUD) is a chronic, relapsing mental and behavioral condition characterized by impaired control over Alcohol consumption, persistent drinking despite adverse consequences, and the development of dependence. The global burden of AUD is substantial. A recent report cited in a 2025 global analysis estimated that 109 million individuals worldwide were diagnosed with AUD in 2019 [1]. Updated data from the Global Burden of Disease Study 2021 indicated that the number of people aged ≥ 15 years with AUD increased from 84.55 million in 1990 to 111.12 million in 2021, while the age-standardized prevalence declined from 1,697.9 to 1,335.4 per 100,000 over the same period (average annual percent change − 0.78%) [2]. Adolescence constitutes a particularly vulnerable developmental window for the initiation of risky drinking behaviors, which can confer long-term neurobiological and behavioral consequences. Beyond its medical impact, AUD imposes considerable social and economic costs, including increased healthcare expenditures, reduced productivity, and family disruption. The pathogenesis of AUD reflects widespread alterations across neural circuits governing reward, stress, and executive control, which interact closely with the hypothalamus [3]. As an integrative hub for stress responses, energy balance, neuroendocrine signaling, and emotional regulation, the hypothalamus is increasingly recognized as a key locus of vulnerability in AUD. Alcohol misuse disrupts hypothalamic–pituitary endocrine function and compromises homeostatic regulation [4].

Mechanistic studies have shown that chronic alcohol exposure provokes a cascade of pathological processes in the hypothalamus. One consistent finding is neuroinflammation, where activation of melanocortin-4 receptor pathways can partially counteract ethanol-induced inflammatory responses and, in turn, limit subsequent alcohol intake [5]. Ethanol also imposes oxidative stress and apoptotic injury on developing hypothalamic neurons, highlighting the intrinsic vulnerability of this region [6]. Beyond these local processes, endocrine dysregulation underscores the broader contribution of the hypothalamus to AUD. Altered activity of the hypothalamic–pituitary–adrenal (HPA) axis is closely tied to dependence maintenance and relapse vulnerability [7].

Collectively, current evidence highlights the hypothalamus as a central node in the neuropathology of AUD, where immune activation, oxidative imbalance, synaptic dysregulation, and endocrine disturbances converge. Despite these insights, systematic investigations into the transcriptomic remodeling of the hypothalamus following chronic alcohol exposure remain scarce. To bridge this gap, the present study employed transcriptome sequencing in combination with qRT-PCR validation in alcohol-exposed adolescent mice. This focus is particularly relevant a developmental period characterized by rapid hypothalamic remodeling, including maturation of the hypothalamic–pituitary–gonadal (HPG) axis and reorganization of neuroendocrine circuitry [8, 9]. These developmental features highlight the hypothalamus as a particularly sensitive target of alcohol during adolescence and provide a strong rationale for investigating transcriptomic alterations at this stage to elucidate neurobiological mechanisms and inform novel therapeutic strategies.

## Materials and methods

### Animals and ethics statement

Male C57BL/6 mice, 30–40 days of age (correspond to adolescence, a developmental stage characterized by rapid hypothalamic remodeling) and weighing 15–20 g, were obtained from the Experimental Animal Center of Jiamusi University. Only males were used in order to minimize variability related to the estrous cycle and fluctuations in circulating sex hormones. The age of each animal was recorded as postnatal day (PND) based on vendor-provided date of birth (DOB) and the calendar date of each procedure (PND = date_procedure – DOB). All mice were housed in a standard laboratory cage (40 × 15 cm) with 50% relative humidity and a 12-hour light/dark cycle (with the lights on from 8 am to 8 pm). Adaptive feeding was performed for 1 week prior to the start of the experiment. All procedures in this experiment were performed in accordance with the National Institutes of Health Guide for the Care and Use of Laboratory Animals and were approved by the University Ethics Committee of Jiamusi University.

### Intermittent alcohol procedures

**Fig. 1:**
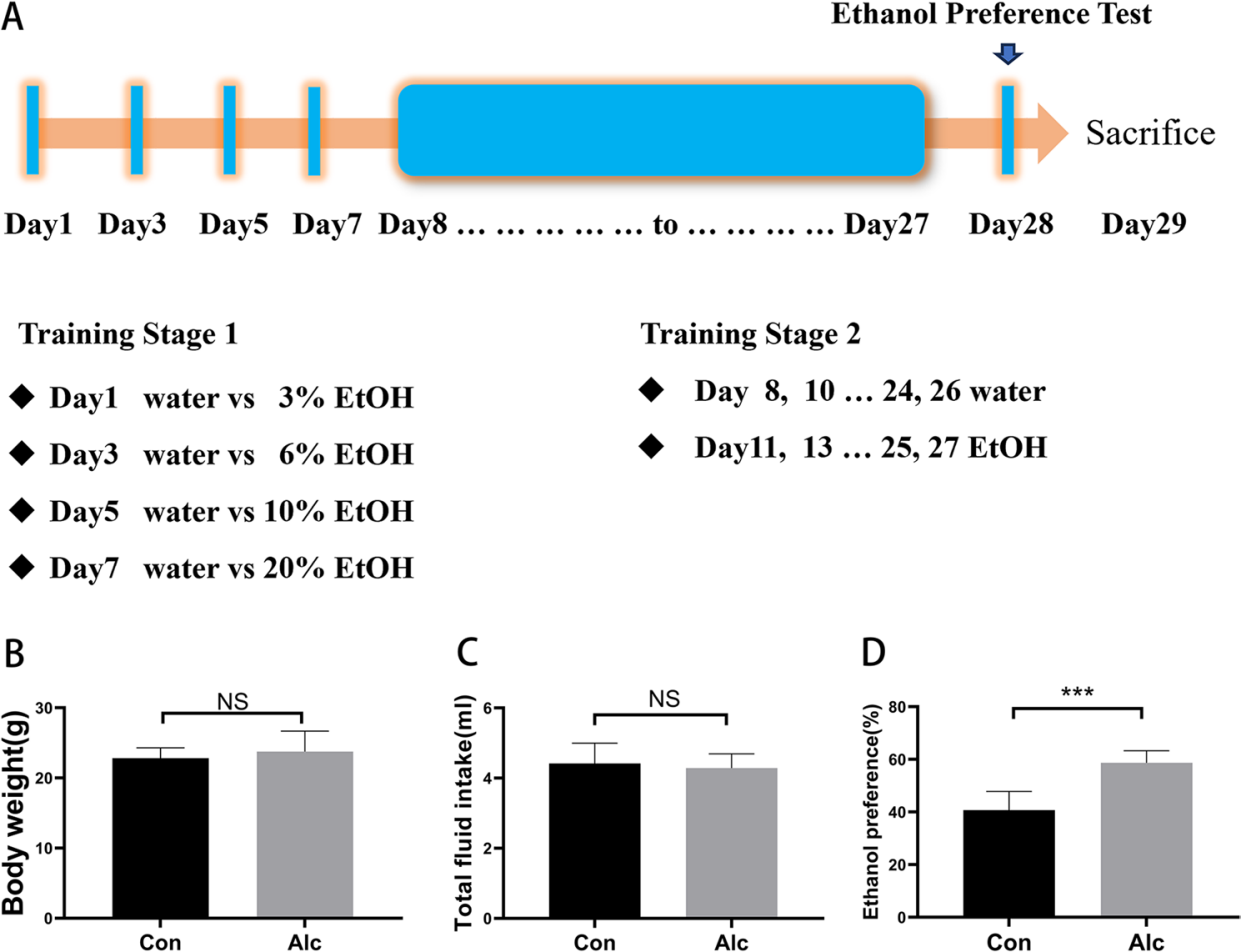
Drinking behavior in mice with alcohol exposure. Comparisons between control and model group mice (*n* = 10). **A**: Schematic of experimental designs. **B**: Body weight; **C**: Total fluid intake; **D**: Alcohol preference. NS: *P* > 0.05; * *P* < 0.05, ** *P* < 0.01, *** *P* < 0.001

A mouse model of alcohol exposure was constructed using two bottles of intermittent free-drinking, as previously described [10]. Alcohol group mice were given two bottles, one containing Alcohol solution and one containing water. The control group was given two bottles of drinking water. The alcohol group was given 3%, 6%, 10%, and 20% (w/v) ethanol solution in the first bottle on day 1, 3, 5, and 7, respectively and water in the second bottle. On the eighth day two bottles of water were given for twenty-four hours. Thereafter, a 20% Alcohol solution was given every other day. The following day the bottle was weighed, washed of ethanol solution, and filled with water. The bottle positions were switched each day to avoid positional favoritism. The Alcohol preference test was performed on the 29th day of the trial and a hypothalamic tissue sample was taken after the test (Fig. 1A).

### Alcohol preference test

The mice were deprived of water for 12 h and then given both an Alcohol solution and water for another 12 h. Ethanol preference is calculated as follows: $$\begin{aligned} &\mathrm{Ethanol}\;\mathrm{preference}\;\left(\%\right)=\mathrm{ethanol}\;\mathrm{intake}\;\left(\mathrm{ml}\right)/\\&\left[\mathrm{ethanol}\;\mathrm{intake}\;\left(\mathrm{ml}\right)+\mathrm{water}\left(\mathrm{ml}\right)\right]\times100\% \end{aligned}$$ 

### Transcriptome sequencing

Total hypothalamic RNA was extracted using the TRIzol method, followed by cDNA synthesis using mRNA enrichment, mRNA fragmentation, cDNA inversion, adapter attachment, fragment screening, and library enrichment on the NovaSeq X Plus platform. Fastp (https://github.com/OpenGene/fastp) was used for data quality control. HiSat2 (http://ccb.jhu.edu/software/hisat2/index.shtml) was used for quality assessment, including determination of gene sequencing saturation coverage, and of regional and chromosomal distribution of reads. RSEM (http://deweylab.github.io/RSEM/) was used for quantitative analysis of transcript expression levels.

### Differential expression analysis and bioinformatic analysis

DESeq2 was used to screen for differentially expressed genes among samples using screening criteria: *P* < 0.05 and |log2FC|≧ 1. Gene ontology (GO) (https://www.geneontology.org/) and Kyoto Encyclopedia of Genes and Genomes (KEGG) (https://www.genome.jp/kegg/) databases were queried for bioinformatic analyses. All analyzed datasets are provided in the Supplementary Materials.​.

### Real-time fluorescence quantitative PCR (qRT-PCR)

For qRT-PCR validation, we selected all differentially expressed genes from the KEGG-enriched GnRH signaling pathway (top-ranked) and the thyroid hormone synthesis pathway (fifth-ranked but biologically relevant to hypothalamic endocrine regulation). The only exception was the predicted gene Gm49320, which was excluded due to lack of functional annotation. RNA was extracted from hypothalamus samples using TRIzol. Reverse transcription was performed using a PrimeScript RT kit, and quantitative PCR was performed using SYBR Premix Ex Taq II (Takara) with *Gapdh* as the internal reference. Differences in mRNA levels were calculated using the 2^−△△Ct^ method. Primers for qRT-PCR were synthesized by Shanghai BioEngineering Co. The primer sequences used for qRT-PCR are provided in Supplementary Table I.

### Statistical analysis

IBM SPSS Statistics 29.0 was used for data processing. Data are given as the mean ± SEM. Differences between the two groups were assessed using a T-test. *P* < 0.05 was considered statistically significant.

## Results

### Alcohol preference in mice with alcohol exposure

A mouse model of alcohol exposure was established according to previous studies. Compared with the control group, the alcohol preference of alcohol exposure mice was significantly increased after intermittent alcohol intake (Fig. 1C, *P* < 0.001). However, there was no significant difference in body weight (Fig. 1A, *P* > 0.05) or total fluid intake (Fig. 1B, *P* > 0.05) between the two groups.

### Differential expression of genes in the hypothalamus of mice with alcohol exposure

**Fig. 2:**
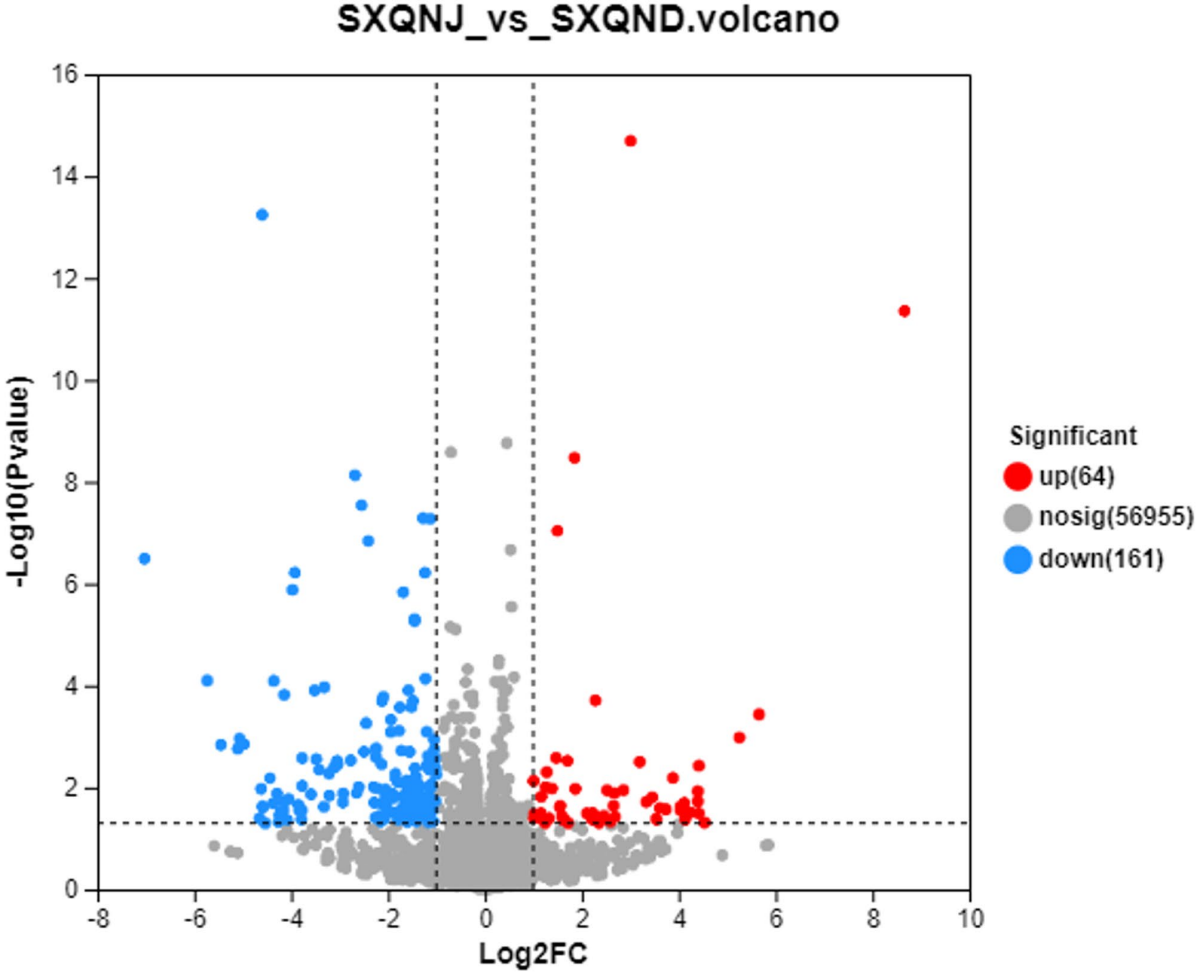
Volcano plot of differentially expressed genes in the hypothalamus of mice with alcohol exposure

Transcriptome sequencing was performed on the hypothalami of alcohol and control group mice. Using cutoff conditions of *P* < 0.05 and | log2FC| ≧ 1 we identified 225 differentially expressed genes between the two groups, of which 64 had increased expression and 161 had decreased expression (Fig. 2).

### Alcohol exposure modulates the expression of hypothalamic development-related genes in mice

**Fig. 3:**
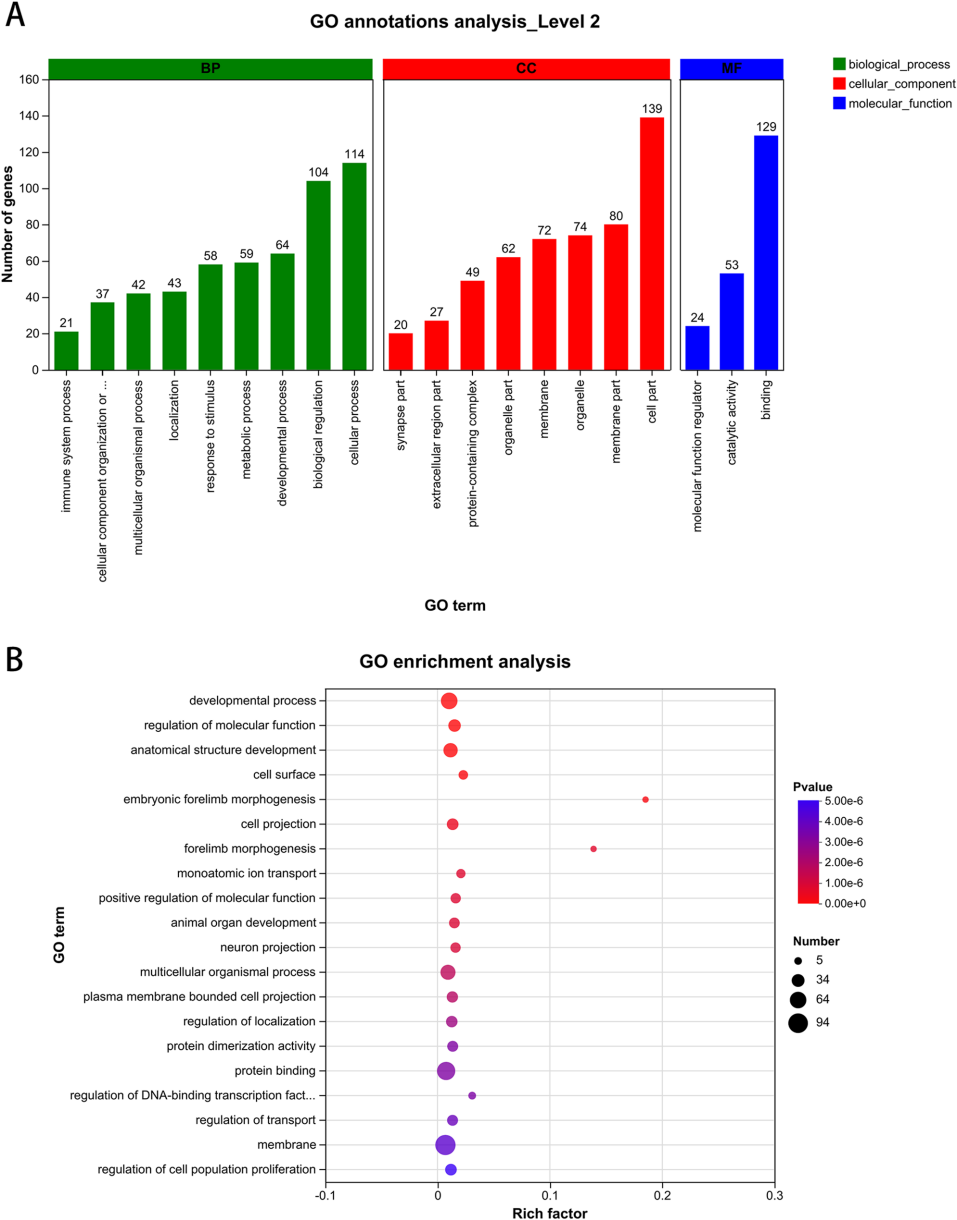
GO analysis of differentially expressed genes in the hypothalamus of mice with alcohol exposure; **A**: GO annotation analysis; **B**: GO enrichment analysis

GO annotation analysis was applied to analyze the differentially expressed genes. The largest numbers of annotated genes were: 114 for the Biological Process (BP) term, cellular process (GO:0009987), 139 for the Cellular Component (CC) term, cell part (GO:0044464), and 129 for the Molecular Function (MF) term, binding (GO:0005488) (Fig. 3A). These results also showed that developmental process (GO: 0032502) had the highest degree of enrichment (*P* < 0.001), with 62 genes enriched, among which 11 genes showed increased expression and 41 genes showed decreased expression. These findings indicate that alcohol exposure modulates the expression of hypothalamic development-related genes in mice.

### Alcohol exposure modulates the expression of GnRH and thyroid-related genes in the hypothalamus

**Fig. 4:**
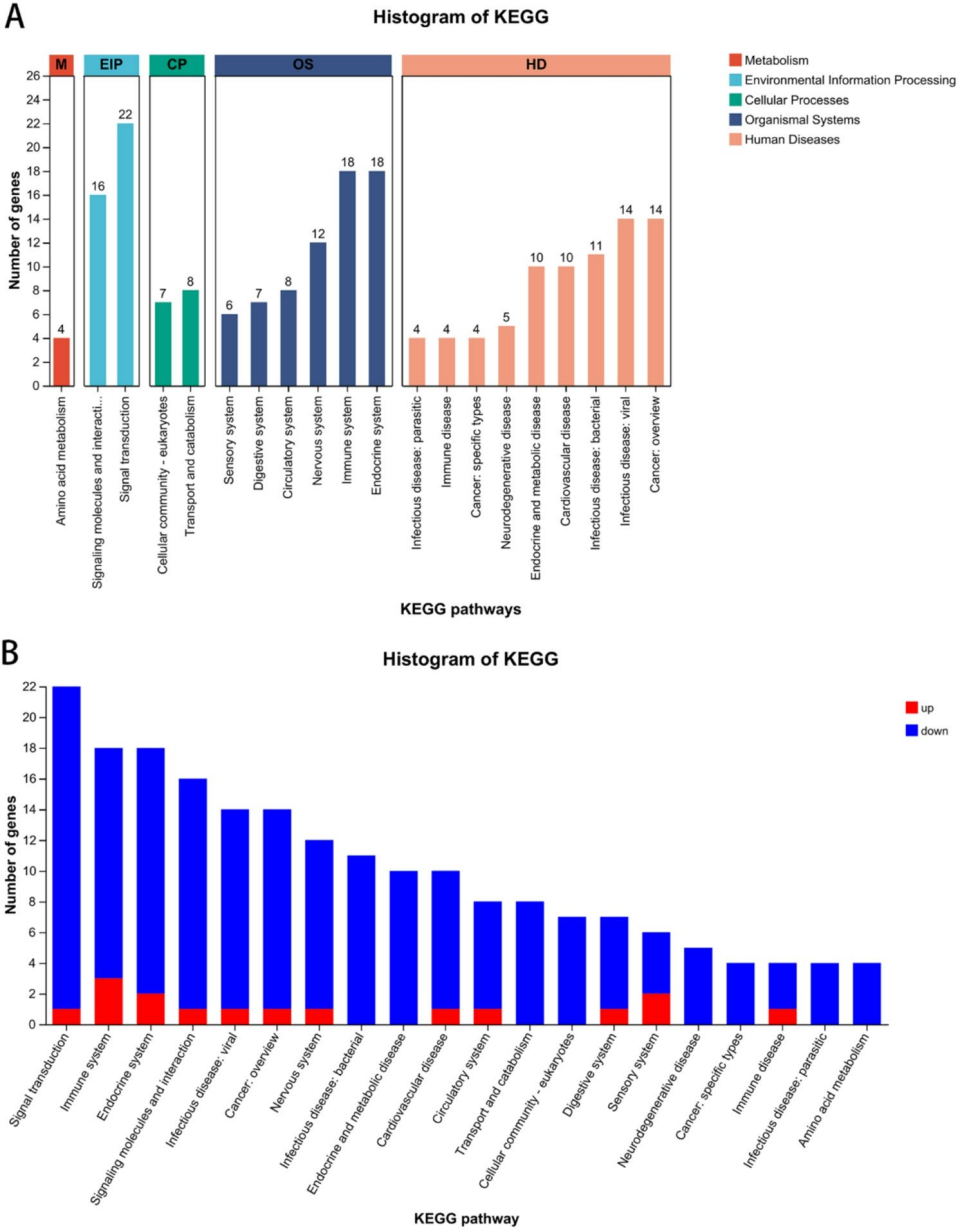
KEGG annotation analysis of differentially expressed genes in the hypothalamus of mice with alcohol exposure; **A**: Column diagram of the KEGG annotation analysis; **B**: Accumulation map of KEGG annotation analysis

**Fig. 5:**
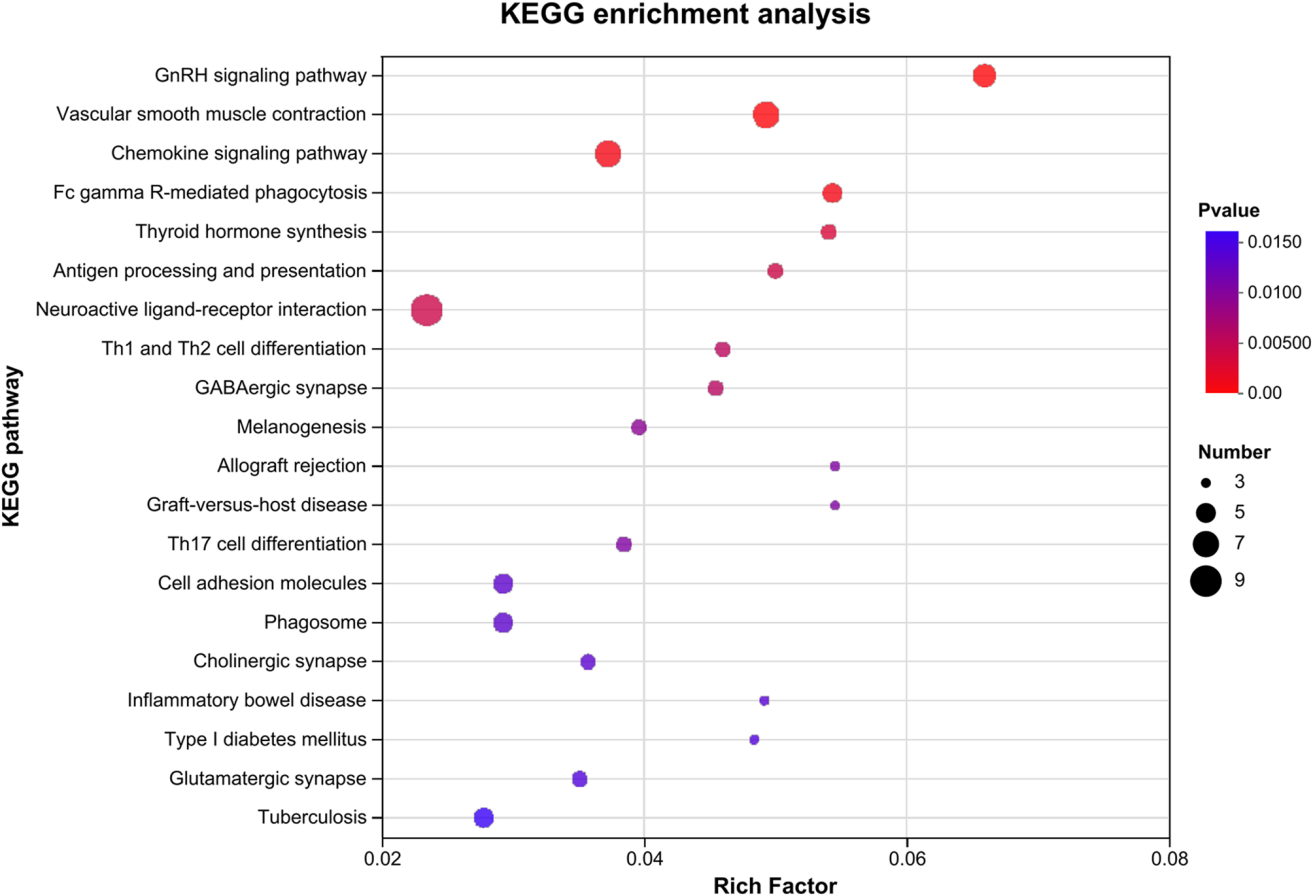
KEGG enrichment analysis of differentially expressed genes in the hypothalamus of mice with alcohol exposure

KEGG annotation analysis of the differentially expressed genes showed that the term, Signal transduction pathway, had the largest number of annotated genes (22 genes) (Fig. 4A). The accumulation map shows the expression of one gene was increased and the expression of 21 genes was decreased (Fig. 4B). The Immune system and Endocrine system terms both have 18 annotated genes (Fig. 4A). For the Immune system genes, the expression of three was increased, and that of 15 was decreased (Fig. 4B). For the Endocrine system genes, the expression of two genes was increased and that of 16 genes was decreased (Fig. 4B). KEGG enrichment analysis showed that the five pathways with the highest enrichment were: GnRH signaling pathway (mmu04912, *P* < 0.001), Vascular smooth muscle contraction (mmu04270, *P* = 0.0002), Chemokine signaling pathway (mmu04062, *P* = 0.0008), Fc gamma R-mediated phagocytosis (mmu04666, *P* = 0.0009), and Thyroid hormone synthesis (mmu04918, *P* = 0.0031) (Fig. 5).

#### Alcohol exposure is associated with decreased expression of GnRH signaling-related genes in the hypothalamus of mice

**Fig. 6:**
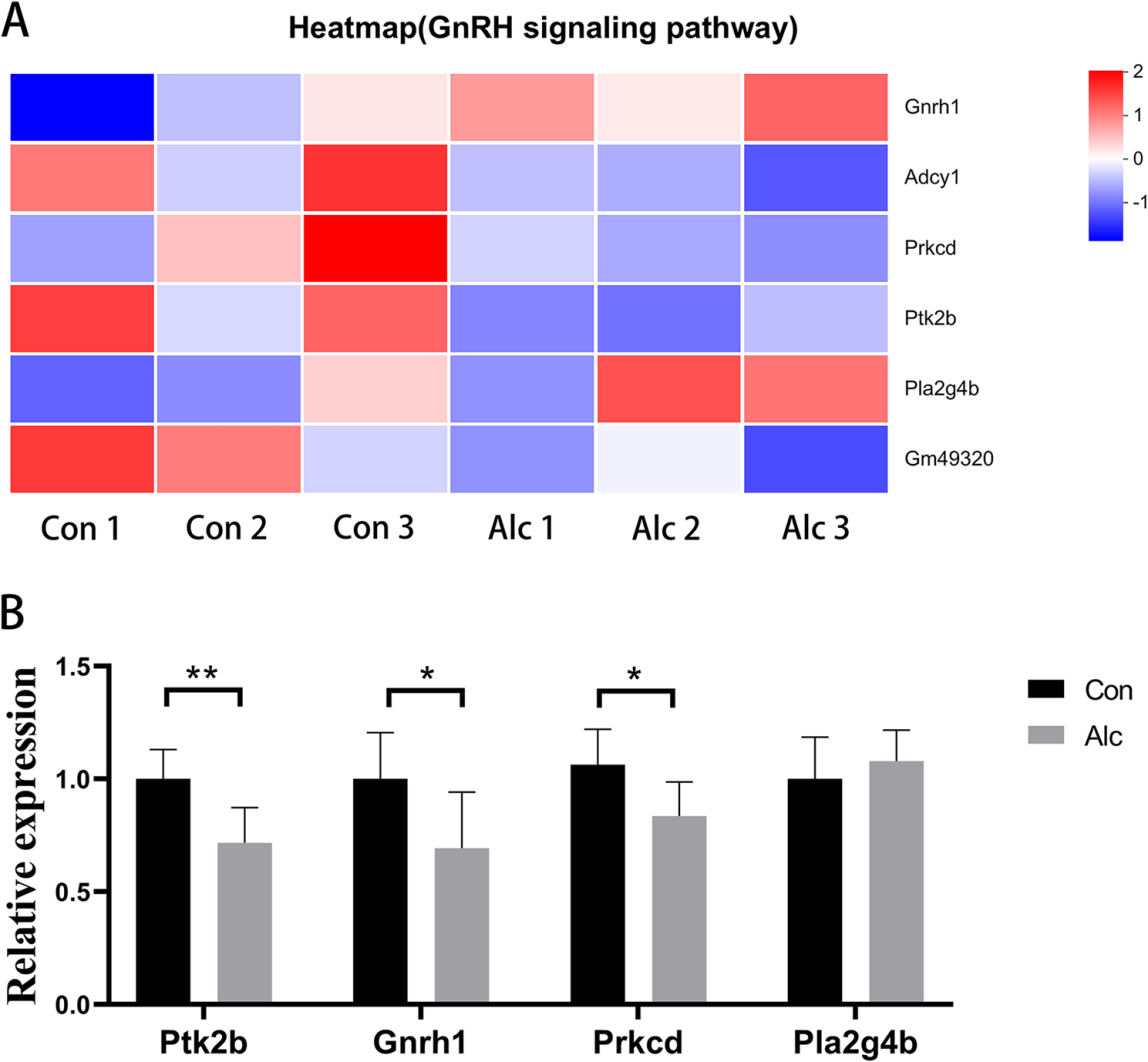
Effects of alcohol exposure on the expression of GnRH signaling-related genes in the hypothalamus of mice; **A**: Heatmap of sequencing results (*n* = 3); **B**: PCR verification (*n* = 6). * *P* < 0.05, ** *P* < 0.01, *** *P* < 0.001

**Fig. 7:**
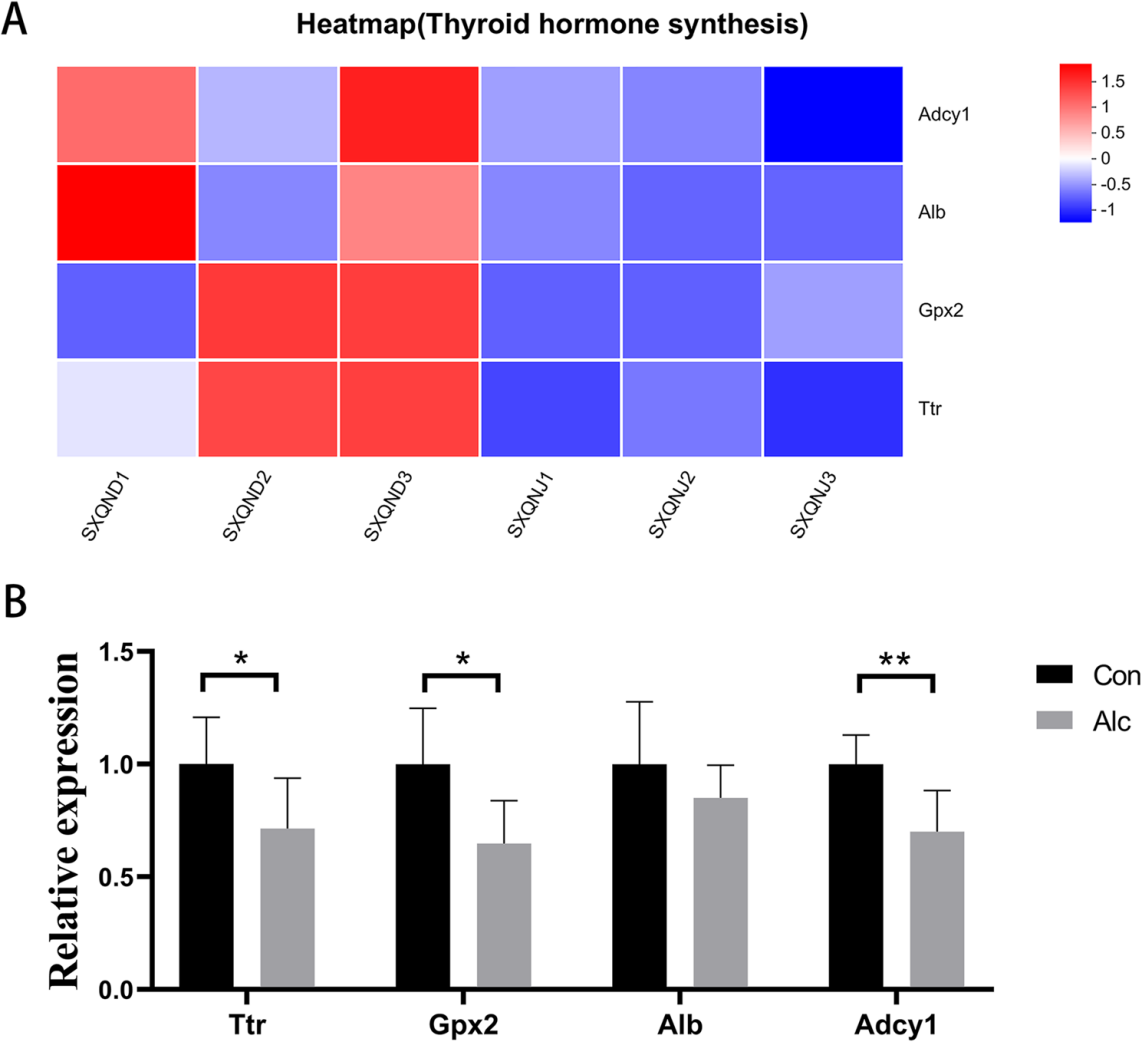
Effects of alcohol exposure on the expression of genes related to the thyroid hormone synthesis pathway in the hypothalamus of mice; **A**: Heatmap of sequencing results (*n* = 3); SXQND1–3 represent Control group samples, and SXQNJ1–3 represent Alcohol group samples. **B**: PCR verification (*n* = 6). * *P* < 0.05, ** *P* < 0.01, *** *P* < 0.001

KEGG enrichment analysis showed that GnRH signaling pathway (mmu04912) had the highest degree of enrichment (*P* < 0.001), with six enriched genes: *Prkcd*, *Gnrh1*, *Adcy1*, *Gm49320*, *Ptk2b*, and *Pla2g4b*. These sequencing results were validated by PCR. Compared with the control group, the expression of *Prkcd* (*P* = 0.028), *Ptk2b* (*P* = 0.007), and *Adcy1* (*P* = 0.008) was decreased in the alcohol group. These results were consistent between sequencing and PCR results (Figs. 6 and 7). Meanwhile, PCR showed that expression of *Gnrh1* (*P* = 0.043) decreased and expression of *Pla2g4b* (*P* = 0.416) was unchanged between the control and alcohol groups, which was inconsistent with the sequencing results (Fig. 6).

#### Alcohol exposure is associated with decreased expression of genes related to the thyroid hormone synthesis pathway in the hypothalamus of mice

KEGG enrichment analysis showed that Thyroid hormone synthesis pathway (mmu04918) was greatly enriched (*P* = 0.0031), with four enriched genes: *Adcy1*, *Gpx2*, *Ttr*, and *Alb*. PCR verification of these four genes showed that compared with the control group, the expression of *Adcy1* (*P* = 0.008), *Gpx2* (*P* = 0.020) and *Ttr* (*P* = 0.045) was decreased in the alcohol group, which was consistent with the sequencing results (Fig. 7). There was no difference in the expression of *Alb* (*P* = 0.269) between the two groups (Fig. 7). These findings suggest that alcohol exposure is associated with reduced expression of thyroid hormone synthesis–related genes in the hypothalamus.

## Discussion

The hypothalamus regulates the endocrine system and the autonomic nervous system. It is involved in the regulation of emotional behavior and energy balance, and maintains the stability of the internal environment through the regulation of physiological processes, such as hormone secretion, the stress response, appetite, and body temperature. AUD affects hypothalamic function in a variety of ways. Long-term alcohol consumption affects the hypothalamic-pituitary-adrenal axis and various hormonal systems, resulting in hormone secretion disorders [11]. AUD can also increase neural reward and punishment circuit responses and can enhance binge drinking behavior and alcohol dependence by altering the hypothalamic-nucleus accumbens pathway [12, 13]. In addition, sustained alcohol intake affects the hypothalamus and the limbic lobe, inducing mood swings, anxiety, and depression-related behavior [14, 15]. AUD also may affect metabolic regulation pathways in the hypothalamus, and disturbs energy metabolism [16]. The combined effect of these changes contributes to the development and persistence of AUD. Here, we used the two-bottle approach to construct a mouse model of Alcohol exposure and then performed transcriptome sequencing in the hypothalamus. GO enrichment analysis showed that the differentially expressed genes were most highly enriched for developmental process terms, with the expression of 11 genes increased and 41 genes decreased. Maternal fetal alcohol exposure can affect fetal brain development and damage brain tissue and it has been proposed that alcohol exposure modulates the expression of hypothalamic development-related genes in mice [17, 18]. Our previous transcriptome sequencing study of astrocytes with moderate alcohol injury also confirmed that alcohol intervention modulates the expression of genes involved in astrocyte development [19]. These results are consistent with the involvement of development-related gene expression in alcohol-associated changes in cellular and brain function.

AUD can affect hypothalamic hormone secretion through a variety of mechanisms. Continuous alcohol intake activates the hypothalamic–pituitary–adrenal (HPA) axis, leading to abnormal secretion of stress-related hormones such as cortisol and thereby exacerbating stress reactivity, anxiety, and depressive symptoms. Evidence from adolescent models indicates that pubertal binge alcohol exposure induces long-lasting alterations in HPA axis reactivity [20], while human studies show that adolescent binge drinking predicts atypical cortisol stress responses in young adulthood [21].These changes in hormone secretion provide the physiological basis for the occurrence and maintenance of Alcohol dependence. In this study, KEGG annotation analysis was performed on differentially expressed genes in the hypothalamus between the Alcohol and control groups. The term, Signal Transduction pathway, had the highest number of annotated genes, with the expression of one gene increasing and 21 genes decreasing. Both Immune System and Endocrine System terms had 18 annotated genes, with the expression of three genes increased and 15 genes decreased for Immune System. For Endocrine System, expression was increased for two genes and decreased for 16 genes. These findings show that alcohol exposure is associated with altered expression of genes related to signal transduction, the immune system, and the endocrine system, with predominantly decreased expression. KEGG enrichment analysis revealed the highest enrichment levels were for the GnRH signaling pathway.

GnRH is a neuropeptide secreted by the hypothalamus that is involved in regulating the function of the reproductive system by stimulating the pituitary gland to secrete gonadotropin-releasing hormones (luteinizing hormone and follicle-stimulating hormone). It is an important regulator of the hypothalamic-pituitary-gonadal axis. GnRH not only regulates the synthesis and secretion of sex hormones, such as testosterone, estrogen, and progesterone through the gonadal axis, but is also closely related to various neuroendocrine functions, such as emotional regulation, and the stress response. GnRH neurons in the hypothalamus are regulated by a variety of factors, including the cortex, the emotional center, endocrine hormones, and additional signaling pathways. The secretion of GnRH is regulated by the external environment and the individual’s psychological state, which can be directly or indirectly affected by emotional changes, stress, and stressful states. Sustained alcohol consumption can significantly inhibit the secretion and function of sex hormones [22]. Alcohol inhibits GnRH secretion by directly inhibiting the function of GNRH neurons in the hypothalamus [22]. In men, GnRH secretion is relatively stable and maintains constant testosterone levels. Sustained alcohol consumption lowers the body’s testosterone levels, which in turn affects sperm production and libido. In addition, the effects of alcohol on sex hormone levels can also interfere with GnRH secretion and gonad function through feedback mechanisms. Here, we identified five genes enriched in the GnRH signaling pathway, all of which showed decreased expression in the alcohol group, indicating that alcohol exposure is associated with reduced expression of GnRH signaling pathway genes.

Protein kinase C delta type (PRKCD) is a member of the Protein kinase C (PKC) family. This calcium-independent, diacylglycerol (DAG)-dependent serine/threonine protein kinase plays an important role in cell development and is a pro-apoptotic factor during apoptosis induced by DNA damage, while it has anti-apoptotic properties during apoptosis mediated by cytokine receptors. Additionally, PRKCD is required for the production of reactive oxygen species by NADPH oxidase. At the same time, PRKCD can negatively regulate B cell proliferation and can play an important role in autoantigen-induced B cell tolerance [23, 24]. During GnRH pathway transduction, GnRH binds to GnRHR to activate the Gq/11 G protein and phospholipase C (PLC). PLC catalyzes the decomposition of phosphatidylinositol 4,5-bisphosphate into DAG and inositol 1,4,5-trisphosphate. DAG then directly activates PKC, thereby regulating the synthesis and secretion of gonadotropins (such as luteinizing hormone and follicle-stimulating hormone). Activated PKC can also directly or indirectly phosphorylate key sites of Protein-tyrosine kinase 2-beta (PTK2B) (such as Tyr402 or other serine/threonine residues), resulting in conformational change and increased PTK2B activity. PTK2B, also known as PYK2, is a non-receptor protein tyrosine kinase involved in actin skeleton recombination, cell polarization, cell migration, adhesion, diffusion and bone remodeling, and plays an important role in regulating the humoral immune response. It is necessary for the normal migration of B cells in the spleen. PTK2B has a FERM region, a tyrosine kinase domain, and multiple sites that can bind to Src homology 2 (SH2)/Src homology 3 (SH3) domain proteins, and may form complexes with PKC or PKC-related proteins [25]. The resulting signaling complex co-regulates downstream targets in the GnRH pathway. Our sequencing results and PCR validation showed a decrease in *Prkcd* and *Ptk2b* mRNA levels in the alcohol group, suggesting that alcohol exposure is associated with reduced expression of *Prkcd* and downstream *Ptk2b*, which may influence GnRH pathway activity in the hypothalamus.

The sequencing and PCR validation results jointly demonstrate that Alcohol exposure can also inhibit the expression of Adenylate cyclase type 1 (*Adcy1*). *Adcy1* is a key node in the cAMP second messenger pathway and is involved in regulating the cAMP/Protein kinase A (PKA) pathway. By activating Ca^2+^/Calmodulin, ATP catalyzes the production of cAMP, which further activates downstream pathways, such as PKA and EPAC, which affect gene transcription, synaptic transmission and neuroplasticity [26]. In brain regions related to AUD (such as the nucleus accumbens, prefrontal cortex, and hippocampus), the cAMP/PKA pathway is involved in the regulation of reward, motivation, and emotion. In the GnRH pathway, GnRH binding to GnRHR can activate ADCY1, which then activates the cAMP/PKA pathway, which is involved in regulating the expression of gonadotropin-related genes. ADCY1 is also involved in transduction of the thyroxine synthesis pathway [27]. After binding to its receptor, thyrotropin can activate ADCY1, which then activates the cAMP/PKA pathway, affecting downstream gene expression and ultimately regulating thyroxine synthesis and secretion. In this study, the level of *Adcy1* mRNA in the hypothalamus decreased after alcohol intervention, indicating that alcohol exposure is associated with decreased Adcy1 expression in the hypothalamus, which may influence both GnRH signaling and the thyroid hormone synthesis pathway.

The KEGG enrichment analysis of this study revealed significant effects of alcohol consumption on the thyroid hormone synthesis pathway. Thyroxine (T3, T4) is secreted by the thyroid gland under the control of the hypothalamic-pituitary-thyroid axis and enters the blood circulation to maintain energy metabolism balance, neurodevelopment, and multi-organ function. AUD interferes with the function of the hypothalamic-pituitary-thyroid axis and induces malnutrition, liver injury, and inflammatory stress. AUD also causes thyroid hormone production and metabolic abnormalities. The initiation and persistence of AUD can be facilitated by changes in thyroxine levels that effect basal metabolic rate, emotional state, and neural excitability. In this study, four genes in the thyroid hormone synthesis pathway were enriched: *Adcy1*, *Gpx2*, *Ttr*, and *Alb*. PCR quantification of for *Adcy1*, *Gpx2*, and *Ttr* mRNA levels were consistent with the sequencing results, with low expression in the Alcohol group, indicating that Alcohol exposure may inhibit the function of the thyroid hormone synthesis pathway. Glutathione peroxidase 2 (GPX2) is a member of the glutathione peroxidase (GPX) family, which mainly participates in intracellular oxidation. GPX2 reduces cellular damage from oxidative stress by catalyzing glutathione (GSH) to reduce peroxides such as H₂O₂ to water or corresponding alcohols [28, 29]. It plays an important antioxidant role to combat the oxidative damage of AUD [30]. In this study, *Gpx2* expression was reduced in the alcohol group, indicating that alcohol exposure promotes oxidative damage in the hypothalamus by inhibiting *Gpx2* expression and thereby affecting hypothalamic function. Transthyretin (Ttr) is a thyroid hormone-binding protein that has the ability to specifically bind thyroxine (mainly T4) and transport it from the blood to the brain. After entering the central nervous system through the blood–brain barrier, T4 binds to Ttr, which protects it from degradation and prolongs its half-life [31, 32]. When thyroxine levels are low or metabolic demands increase, Ttr increases thyroxine release to maintain local concentration balance. The reduced levels of *Ttr* mRNA in the alcohol group suggest that alcohol exposure may affect the local balance of thyroxine through decreased hypothalamic *Ttr* expression. This inhibition may also affect the negative feedback mechanism of thyroxine in the hypothalamus, and further affect the function of the hypothalamic-pituitary-thyroid axis and the synthesis and secretion of thyroxine.

In this study, an alcohol exposure mouse model was constructed and transcriptome sequencing was performed to analyze changes in hypothalamic transcription levels. GO analysis revealed that alcohol exposure in mice modulates the expression of genes associated with hypothalamic development. KEGG analysis revealed that alcohol exposure inhibited the expression of genes associated with the GnRH signaling pathway and the thyroid hormone synthesis pathway. PCR confirmed that alcohol exposure was associated with decreased expression of the GnRH signaling pathway genes, *Prkcd* and *Ptk2b*, and the thyroid hormone synthesis pathway genes, *Gpx2* and *Ttr*, and of *Adcy1*, which is shared by both pathways. These alterations may reflect adaptive or maladaptive responses to alcohol exposure, and their precise role in the development of AUD requires further investigation.

### Limitations

Notably, the expression patterns of *Alb*, *Gnrh1* and *Pla2g4b* differed between RNA-seq and qRT-PCR. Such discrepancies may reflect methodological differences in detection sensitivity and isoform coverage, as well as biological variability associated with low abundance, cell-type specificity, or post-transcriptional regulation. While these limitations should be acknowledged, the overall concordance observed for the other validated genes supports the robustness of our transcriptomic dataset, and further validation with larger cohorts and functional assays will be necessary. Moreover, as only male mice were included in this study, sex-specific differences in hypothalamic transcriptomic responses could not be evaluated and should be systematically investigated in future work. However, because the hypothalamus undergoes substantial remodeling during adolescence, it remains possible that some transcriptomic alterations reflect the interaction between alcohol exposure and ongoing maturational processes. Future studies including both adolescent and adult cohorts will be necessary to distinguish developmental from alcohol-specific effects.

## Supplementary Information

Supplementary Material 1.

Supplementary Material 2.

Supplementary Material 3.

Supplementary Material 4.

Supplementary Material 5.

## Data Availability

The datasets supporting the conclusions of this article are included within the article and its Additional files.

## Abbreviations

AUD: Alcohol use disorder
GO: Gene Ontology
KEGG: Kyoto Encyclopedia of Genes and Genomes
GnRH: Gonadotropin-releasing hormone
Prkcd: Protein kinase C delta type
Ptk2b: Protein-tyrosine kinase 2-beta
Adcy1: Adenylate cyclase type 1
Gpx2: Glutathione peroxidase 2
Ttr: Transthyretin
