# Supplementary material for: Alcohol exposure significantly influences gene expression in the hypothalamus, highlighting complex links with gonadotropin-releasing hormone signaling and thyroid hormone production in adolescent mice
Source: BMC Med Genomics. 2025 Oct 7;18:152. doi: 10.1186/s12920-025-02235-z (PMC12506331; doi:10.1186/s12920-025-02235-z)
Supplement: Supplementary file 5 — Supplementary Material 5. [file 12920_2025_2235_MOESM5_ESM.docx]

Table I. Primer sequences used for qRT-PCR validation.

| **Gene name** | **Sequence**(5'→ 3') | **Length** | **Location** |
| --- | --- | --- | --- |
| Prkcd | F: CCTCCTGTACGAAATGCTCATC | 22 | 1602-1623 |
|  | R: GTTTCCTGTTACTCCCAGCCT | 21 | 1782-1762 |
| Gnrh1 | F: AGCACTGGTCCTATGGGTTG | 20 | 65-84 |
|  | R: GGGGTTCTGCCATTTGATCCA | 21 | 169-149 |
| Adcy1 | F: GTCACCTTCGTGTCCTATGCC | 21 | 502-522 |
|  | R: TTCACACCAAAGAAGAGCAGG | 21 | 671-651 |
| Ptk2b | F: TGAGCCCTTGAGCCGTGTA | 19 | 15-33 |
|  | R: AGCTTGAAGTTCTTCCCTGGG | 21 | 173-153 |
| Pla2g4b | F: TGGCCCCTAGCCAACTTTG | 19 | 1142-1160 |
|  | R: GTTCTGGCCTCGACTCAGG | 19 | 1314-1296 |
| Alb | F: TGCTTTTTCCAGGGGTGTGTT | 21 | 45-65 |
|  | R: TTACTTCCTGCACTAATTTGGCA | 23 | 211-189 |
| Gpx2 | F: GCCTCAAGTATGTCCGACCTG | 21 | 254-274 |
|  | R: GGAGAACGGGTCATCATAAGGG | 22 | 396-375 |
| Ttr | F: TTGCCTCGCTGGACTGGTA | 19 | 27-45 |
|  | R: TTACAGCCACGTCTACAGCAG | 21 | 151-131 |
| Gapdh | F: AGGTCGGTGTGAACGGATTTG | 21 | 8-28 |
|  | R: TGTAGACCATGTAGTTGAGGTCA | 23 | 130-108 |
